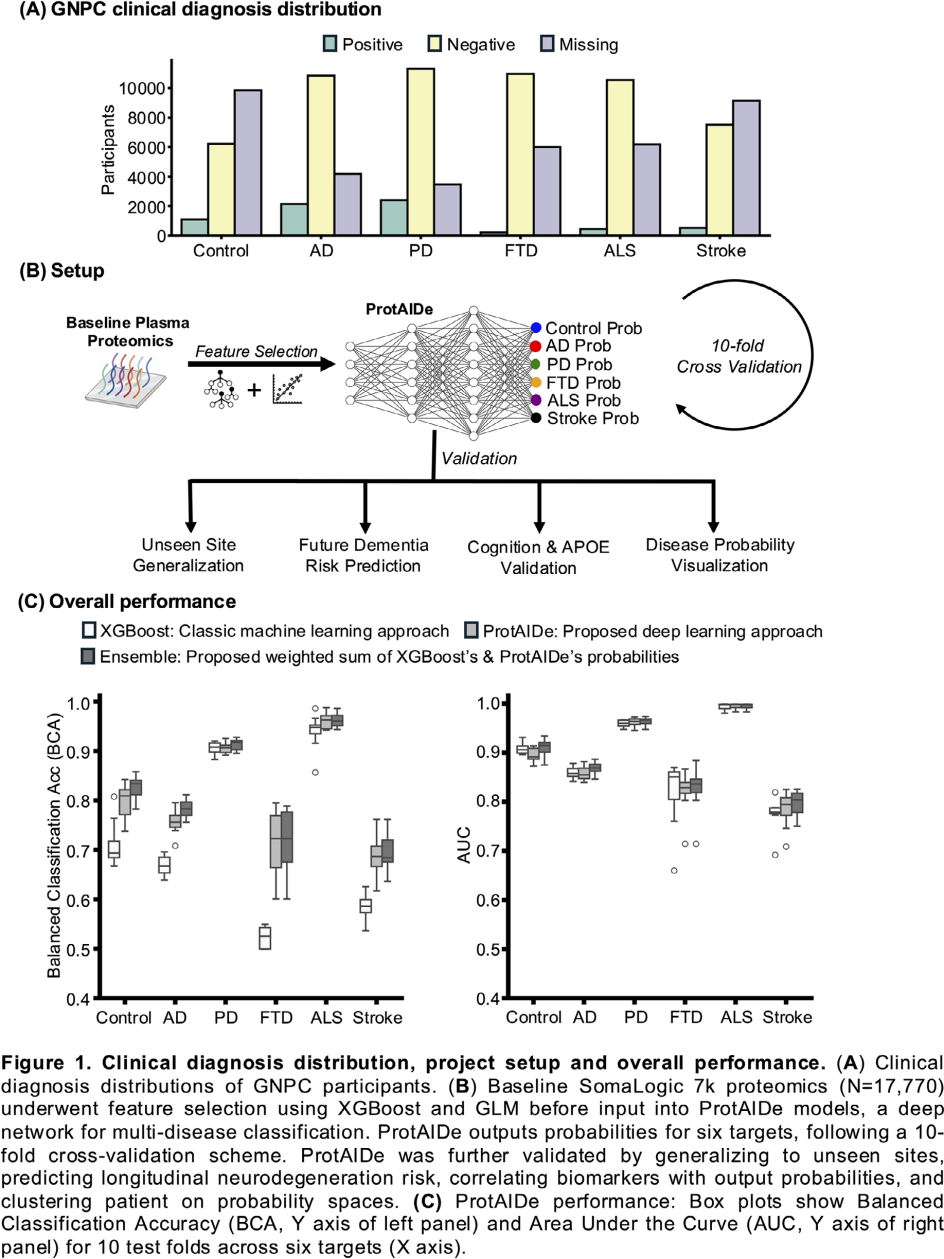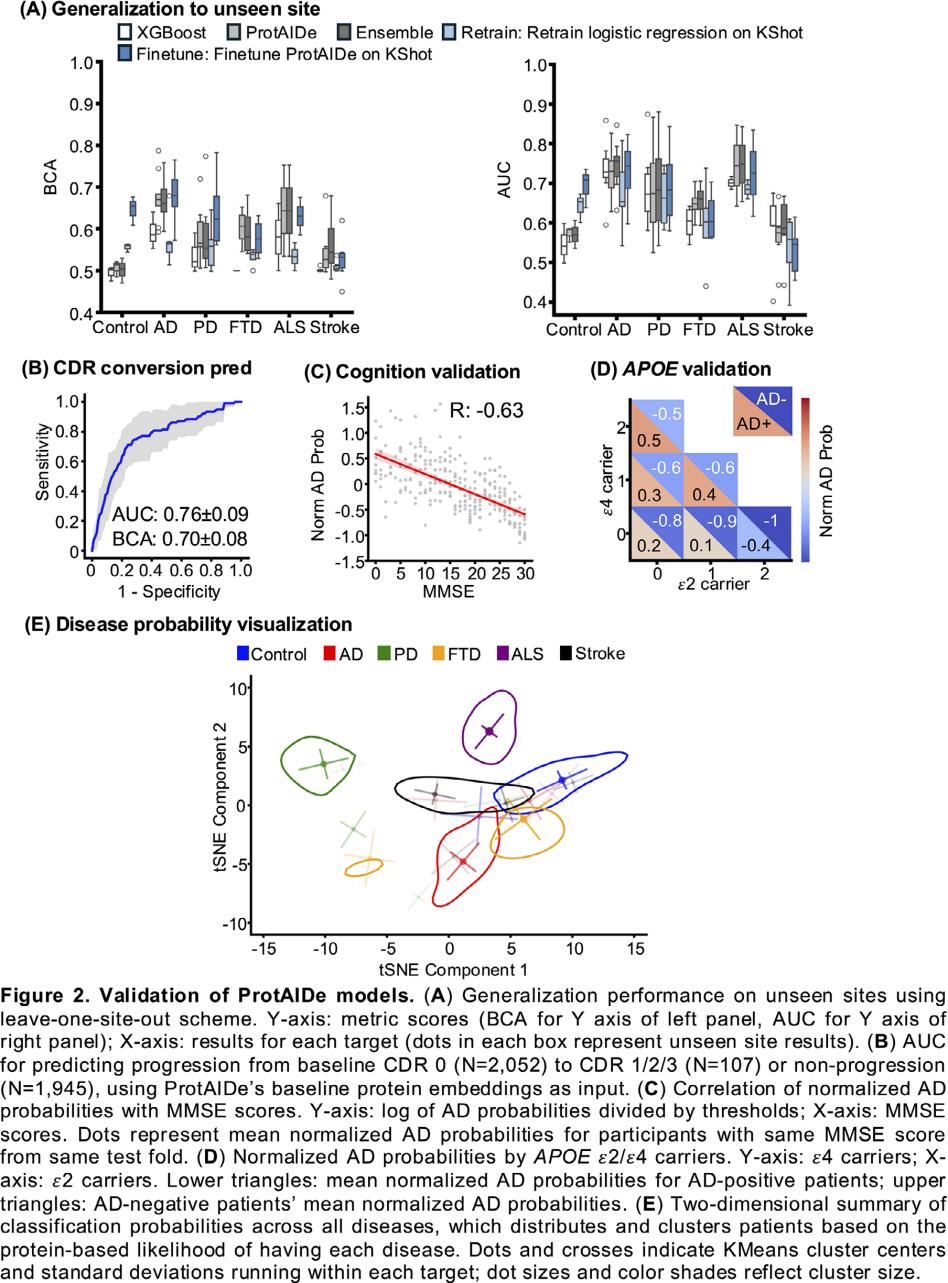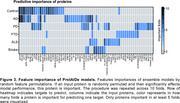# Benchmarking the AI‐based diagnostic potential of plasma proteomics for neurodegenerative disease in 17,710 people

**DOI:** 10.1002/alz70856_099373

**Published:** 2025-12-24

**Authors:** Lijun An, Yu Xiao, Ines Hristovska, Shinya Tasaki, Bart Smets, Ying Xu, Varsha Krish, Farhad Imam, Erik Stomrud, Shorena Janelidze, Sebastian Palmqvist, Alexa Pichet Binette, Rik Ossenkoppele, Niklas Mattsson‐Carlgren, Oskar Hansson, Jacob W. Vogel

**Affiliations:** ^1^ Department of Clinical Sciences Malmö, SciLifeLab, Lund Univerisity, Lund, Sweden; ^2^ Department of Clinical Sciences Malmö, SciLifeLab, Lund University, Lund, Sweden; ^3^ Clinical Memory Research Unit, Department of Clinical Sciences, Lund University, Lund, Sweden; ^4^ Rush Alzheimer's Disease Center, Rush University Medical Center, Chicago, IL, USA; ^5^ Janssen Pharmaceutica NV, a Johnson & Johnson company, Beerse, Belgium; ^6^ Department of Psychiatry, Washington University School of Medicine, St. Louis, MO, USA; ^7^ NeuroGenomics and Informatics Center, Washington University School of Medicine, St. Louis, MO, USA; ^8^ Gates Ventures, Seattle, WA, USA; ^9^ Memory Clinic, Skåne University Hospital, Malmö, Skåne, Sweden; ^10^ Clinical Memory Research Unit, Department of Clinical Sciences Malmö, Lund University, Lund, Sweden; ^11^ Clinical Memory Research Unit, Department of Clinical Sciences Malmö, Faculty of Medicine, Lund University, Sweden, Lund, Sweden; ^12^ Department of Physiology and Pharmacology, Université de Montréal, Montréal, QC, Canada; ^13^ Clinical Memory Research Unit, Department of Clinical Sciences Malmö, Lund University, MOntreal, QC, Canada; ^14^ Centre de recherche de l'institut universitaire de gériatrie de Montréal (CRIUGM), Montréal, QC, Canada; ^15^ Amsterdam Neuroscience, Neurodegeneration., Amsterdam, Netherlands; ^16^ Alzheimer Center Amsterdam, Neurology, Vrije Universiteit Amsterdam, Amsterdam UMC location VUmc, Amsterdam, Netherlands; ^17^ Clinical Memory Research Unit, Lund University, Lund, Sweden; ^18^ Clinical Memory Research Unit, Lund University, Malmö, Skåne, Sweden; ^19^ Memory Clinic, Skåne University Hospital, Malmö, Sweden; ^20^ Department of Clinical Sciences Malmö, Faculty of Medicine, SciLifeLab, Lund University, Lund, Sweden

## Abstract

**Background:**

Plasma proteomic biomarkers have shown significant potential for accurate and cost‐effective dementia diagnosis. However, plasma proteomic has not been validated for multi‐disease diagnosis in large neurodegenerative cohorts yet. This study develops AI models on data from the Global Neurodegeneration Proteomics Consortium (GNPC) to predict major forms of dementia, accounting for multiple underlying pathologies, and outputting probabilistic information.

**Method:**

This study included 17,170 GNPC participants with SomaLogic 7K plasma proteomics, comprising controls and patients with AD, PD, FTD, ALS, or Stroke (Figure 1A). We describe ‘ProtAIDe’, a deep network for six clinical diagnostic categories classification using proteomics (Figure 1B). 10‐fold cross‐validation with built‐in feature selection was used to evaluate overall performance. Leave‐one‐site‐out scheme, with and without k‐shot fine‐tuning, was adopted to evaluate out‐of‐site generalization performance. Additionally, baseline proteomics embeddings from ProtAIDe's last layer were leveraged to predict longitudinal CDR progression (advancing from CDR 0 to >0). Predicted probabilities were validated against cognition and *APOE* genotype, and the cross‐disease classification probability space was visualized using 2‐dimensional t‐SNE. Predictive importance of individual proteins was estimated by feature permutation.

**Result:**

ProtAIDe achieved simultaneous balanced classification accuracy (BCA) >0.7 and AUC >0.8 across all six targets (Figure 1C) with ∼200 proteins. Performance dipped when generalizing to unseen sites, though it was partially recovered by finetuning (Figure 2A). Despite being trained only on baseline data, ProtAIDe's baseline embeddings predicted longitudinal CDR progression with an AUC of 0.76±0.09 (*N* = 2052; Figure 2B). AD classification probability showed a strong anti‐correlation (*R* = ‐0.63) with MMSE score (Figure 2C) and reflected the protective and risk qualities of the *APOE* ɛ2 and ɛ4 alleles, respectively (Figure 2D). t‐SNE visualization of probability spaces (Figure 2E) revealed unique (multiple PD and FTD clusters) and shared (middle regions shared by AD, Control, FTD, and Stroke) clustering patterns among neurodegenerative diseases, suggesting potential comorbidities. Key proteins emerged as major contributors to single‐ or multi‐disease classifications (Figure 3).

**Conclusion:**

Our results demonstrate that ∼200 key proteins in blood can differentiate major forms of dementia at the patient‐level. ProtAIDe's probabilistic information highlights its potential for identifying co‐pathologies and determining proteins driving symptoms at the individual‐level.